# Wearable Cardioverter-Defibrillator Used as a Telemonitoring System in a Real-Life Heart Failure Unit Setting

**DOI:** 10.3390/jcm10225435

**Published:** 2021-11-22

**Authors:** Christian Blockhaus, Stephan List, Hans-Peter Waibler, Jan-Erik Gülker, Heinrich Klues, Alexander Bufe, Melchior Seyfarth, Buelent Koektuerk, Dong-In Shin

**Affiliations:** 1 Heart Centre Niederrhein, Department of Cardiology, Helios Clinic Krefeld, 47805 Krefeld, Germany; stephan.list@helios-gesundheit.de (S.L.); hans-peter.waibler@helios-gesundheit.de (H.-P.W.); heinrich.klues@helios-gesundheit.de (H.K.); alexander.bufe@helios-gesundheit.de (A.B.); buelent.koektuerk@helios-gesundheit.de (B.K.); dong-in.shin@helios-gesundheit.de (D.-I.S.); 2 Witten-Herdecke University, 58455 Witten, Germany; janguelker@gmx.de (J.-E.G.); melchior.seyfarth@helios-gesundheit.de (M.S.); 3 Department of Cardiology, Petrus Hospital Wuppertal, 42283 Wuppertal, Germany; 4 Department of Cardiology, Helios University Hospital, 42117Wuppertal, Germany

**Keywords:** wearable cardioverter-defibrillator, sudden cardiac death, heart failure unit, telemonitoring

## Abstract

Background: In patients with reduced left ventricular ejection fraction (LVEF) who are at risk of sudden cardiac death, a wearable cardioverter-defibrillator (WCD) is recommended as a bridge to the recovery of LVEF or as a bridge to the implantation of a device. In addition to its function to detect and treat malignant arrhythmia, WCD can be used via an online platform as a telemonitoring system to supervise patients’ physical activity, compliance, and heart rate. Methods: We retrospectively analyzed 173 patients with regard to compliance and heart rate after discharge. Results: Mean WCD wearing time was 59.75 ± 35.6 days; the daily wearing time was 21.19 ± 4.65 h. We found significant differences concerning the patients’ compliance. Men showed less compliance than women, and younger patients showed less compliance than patients who were older. Furthermore, we analyzed the heart rate from discharge until the end of WCD prescription and found a significant decrease from discharge to 4, 8, or 12 weeks. Conclusion: WCD can be used as a telemonitoring system to help the involved heart failure unit or physicians attend to and adjust the medical therapy. Furthermore, specific patient groups should be educated more intensively with respect to compliance.

## 1. Introduction

In patients with reduced left ventricular ejection fraction (LVEF) due to structural myocardial disease, cardiac arrhythmias account for a substantial amount of sudden cardiac deaths (SCDs) [1,2]. In this population, coronary artery disease (CAD, 80%) and hypertrophic cardiomyopathy, as well as dilated cardiomyopathy (DCM, 10–15%), are the leading causes of impaired myocardial function with potential arrhythmic complications [3]. The implantable cardioverter defibrillator (ICD) has become an integral component for the primary and secondary prevention of SCD [4]. In patients who do not meet the criteria for primary ICD implantation, but who are at risk of SCD, e.g., due to transient reduced LVEF, the use of wearable cardioverter defibrillators (WCD) is recommended for the period between the acute event or initial diagnosis and the reevaluation after a sufficient time of optimal medical therapy, when the recovery of LVEF is expected or possible [4]. Furthermore, WCD is recommended as a bridge to implantation in patients with existing ICD indications who cannot receive the device or where an existing device has to be extracted due to temporary disorders, such as local or systemic infections [5,6,7,8]. Many studies suggest that using a WCD significantly reduces all-cause mortality in patients at high risk of SCD [9,10,11,12]. However, in a randomized VEST trial including more than 2000 patients, the use of a WCD showed a reduction in SCD but without statistical significance [13]. Furthermore, the device has been proven to detect non-life-threatening cardiac arrhythmias, such as non-sustained ventricular tachycardia (nsVT) or atrial fibrillation (AF) [11]. For the appropriate detection of arrhythmias, it is essential that patients wear the WCD all day and every day, with minimal interruptions. Many studies report wearing compliance of more than 20 h per day [14,15]. Interestingly, the daily wearing time in the VEST trial was considerably below 20 h per day, and many of the documented deaths in this study occurred in patients who did not wear the WCD [13]. Recently, Cheung et al. published a review of evidence and indications of WCD [16].

Patients with symptomatic heart failure, an LVEF less than 35%, and a resting heart rate above 70 beats per minute (bpm) in sinus rhythm and under beta-blocker medication should receive additional treatment with ivabradine, which has been shown to reduce cardiovascular mortality and hospitalization in patients with symptomatic congestive heart failure [4,17,18]. Since medical contact and follow-up infrequently occur in patients even shortly after hospital discharge, continuous monitoring options and remote monitoring systems are gaining more attention as a way to customize medical therapies [19,20]. In this retrospective study, we investigated the daily WCD wearing time in different patient groups and evaluated the potential of heart rate monitoring via an online platform.

## 2. Materials and Methods

The LifeVest^®^ 4000 Model (LifeVest system, ZOLL, Pittsburgh, PA, USA) consists of a tight-fitting electrode belt with two front–back and side–side non-adhesive capacitive dry tantalum oxide electrodes as well as three self-gelling defibrillation electrodes—two posterior and one lateral. The electrodes are connected to the monitor unit, where signals are processed by a rate- and morphology-based detection algorithm. After detection and confirmation of ventricular tachycardia (VT) or ventricular fibrillation (VF), the device sounds a series of alarms before a biphasic shock up to 150 Joule is delivered. During the alarm sequence, shock delivery can be avoided by the patient by pressing the response button [21]. For telemonitoring purpose, the online platform “LifeVest^®^ network” is provided by the manufacturer and can be accessed via a password secured by the prescribing physician. The system automatically tracks all potentially life-threatening arrhythmias, interventions (i.e., alarms and shock delivery), and continuous information, such as heart rate, level of physical activity, and wearing compliance counted in hours per day (h/d).

We retrospectively analyzed 200 consecutive patients who were prescribed a WCD between 2016 and 2020 in our hospital. All patients were initially admitted to the department of cardiology/heart failure unit via our emergency department. The treatment of the primary disease was performed according to the current guideline recommendations. Patient medical history was analyzed with respect to existing or newly diagnosed comorbidities, such as CAD, arterial hypertension, history of stroke, diabetes mellitus II, sleep apnea, chronic obstructive pulmonary disease (COPD), or history of AF. Initial LVEF was determined by transthoracic electrocardiography (TTE) or magnetic resonance imaging (MRI). Follow-up treatment after discharge was carried out by outpatient cardiologists and general practitioners or our outpatient clinic. All patients were offered a follow-up in our outpatient clinic 2–3 months after discharge, with a reevaluation of LVEF and indication for continued use of WCD or implantation of an ICD or cardiac resynchronization therapy defibrillator (CRT-D). Resting heart rate was measured on the day of discharge; follow-up heart rate was determined as a mean value per week by the WCD. Data on heart rate and the time of daily wear (h/d) were accessible via an online-based, password-secured monitoring system that received the data via real-time transmission directly from the devices. The study design was approved by the Ethics Committee of Ärztekammer Nordrhein, Germany (No. 2020014), and the study conformed to the standards defined in the Helsinki Declaration.

Statistical analysis: Categorical data were described by frequencies; continuous data were described by mean, standard deviation, median, and interquartile range. The exact Fisher test was used to test for the independence of two categorical variables. Whether two independent groups differed with respect to the distribution of a continuous variable was tested with the two-sample Wilcoxon rank-sum test. For the comparison of three groups, the Kruskal–Wallis test was used. In case of a significant difference, groups were compared pairwise. The Wilcoxon signed-rank test was used to compare the distribution of continuous data of two time points. Whether WCD compliance was associated with gender, age, or body mass index (BMI) was analyzed by linear regression.

All statistical tests were two-sided at a significance level of 0.05. Statistical analyses were performed using Stata/IC 16.1 for Unix (StataCorp 4905 Lakeway Drive, College Station, TX 77845, USA).

## 3. Results

Between 2016 and 2020, 200 consecutive patients who had an indication and prescription for WCD were included. Indications for WCD prescription consisted mainly of reduced LVEF due to ischemic cardiomyopathy (ICM, group 1), DCM (group 2), or acute inflammatory myocardial disease with (ns)VTs or others, such as the infection of a device, or nsVTs emanating from premature ventricular complexes when ablation was not promptly possible or after ablation for a follow-up time (group 3). Concerning group 3, the indication for WCD mostly consisted of the occurrence of nsVTs in patients with myocarditis and most patients who had an LVEF above 35%. Group 1 consisted of 79 (46%), group 2 of 65 (38%), and group 3 of 29 (17%) patients. In 153 (76.5%) patients, the indicating diagnosis was diagnosed for the first time. Clinically, more than 60% of the patients showed a functional capacity, according to New York Heart Association (NYHA) classes III and IV.

Of the 200 patients included, 1 refused WCD, 1 died of unknown cause(s), 10 patients did not wear WCD at all, and 15 were lost during follow-up. The remaining 173 patients could be enrolled for statistical analysis. The overall acceptance of WCD prescription by health insurance was 96% in 2020. Baseline characteristics of these patients are listed in Table 1. The mean age was 56.64 ± 14.46 years; 45 (26.01%) were females. The mean LVEF for all groups was 28.88 ± 10.45%; NYHA status was class I or II in 66 (38%) patients and class III or IV in 107 (62%) patients.

At discharge, guideline-directed medication was applied in the majority of patients, as shown in Table 2. Beta-blocking agents were prescribed for all patients in groups 1 and 2 and for 86% of group 3. Heart failure therapy with either angiotensin-converting enzyme inhibitors (ACE-I), angiotensin-1 receptor antagonists (AT), or an AT/neprilysin inhibitor was implemented in 98% of patients with ICM, in all patients with DCM, and in 79% of patients with group 3 indications for WCD. Oral anticoagulation was part of the therapy in 64 (37%) patients; a major indication was AF in 56 patients, whereas in 8 patients, left ventricular thrombus was diagnosed by MRI or TTE.

Patients had been instructed to wear the WCD permanently and with the least possible number of interruptions. During a mean WCD wearing time of 59.75 ± 35.6 days with a mean daily wearing time (for all patients) of 21.19 ± 4.65 h, VT was detected and successfully treated by shock delivery in one patient (0.58%). NsVTs with a duration > 10 s were detected in three patients (1.73%). One patient (0.58%) received an inappropriate shock delivery due to tachycardiac AF. Follow-up presentation of patients for re-evaluation of LVEF and determination of the further treatment was scheduled three months after the hospital stay. The documented LVEF significantly improved for all patients compared with baseline EF (38.94 ± 10.64 vs. 28.88 ± 10.45%, *p* < 0.001) but also for all subgroups (group 1: 37.72 ± 9.68 vs. 29.16 ± 7.8%, *p* < 0.001; group 2: 35.81 ± 8.46 vs. 32.57 ± 5.58%, *p* < 0.001; group 3: 49.21 ± 11.59 vs. 40.03 ± 15.68%, *p* < 0.001). ICD or CRT-D was implanted in group 1 in 20.24%, 26.58% in group 2, and 20.69% in group 3. In group 3, the recovery of LVEF was mostly explained by the relatively high number of patients with acute myocarditis with an efficient healing process.

**Figure 1:**
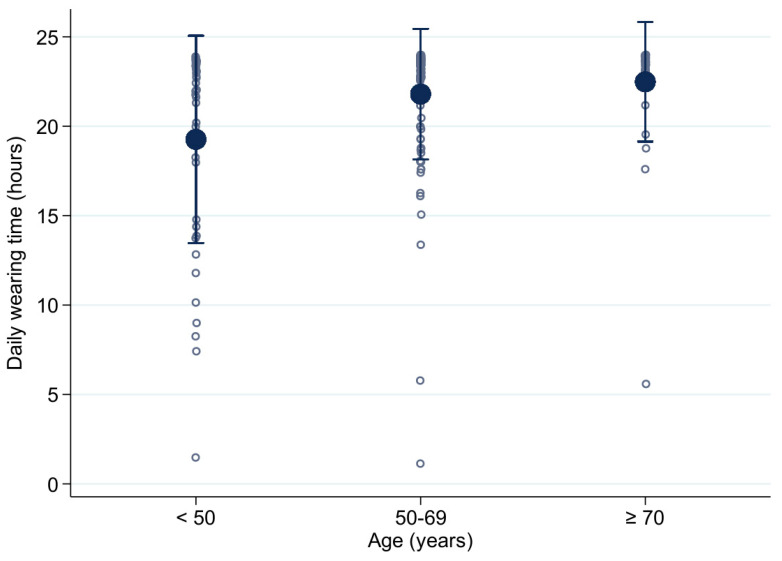
Compliance of different age groups. ° = individual patient data.

Concerning the daily wear time of the WCD, we found significantly better compliance in patients older than 70 years (22.46 ± 3.34 h per day) compared with patients 50–69 years old (21.79 ± 3.65 h per day; *p* = 0.032) or patients younger than 50 years old (18.81 ± 6.45 h per day; *p* = 0.001, Figure 1). Follow-up data are shown in Table 3.

Furthermore, women showed better compliance than men (22.84 ± 1.93 vs. 20.64 ± 5.14 h/d, *p* = 0.01). With regard to body weight, patients with obesity—meaning a BMI of more than 30—showed a trend towards less compliance than patients with normal weight or overweight (20.23 ± 5.77 vs. 21.62 ± 4 h, *p* = 0.185). Furthermore, patients with a BMI of more than 35 showed a trend to reduced compliance (19.8 ± 6.2 vs. 21.6 ± 3.9 h, *p* = 0.159).

Multivariate analysis performed with regard to sex, age, and BMI showed significant results for sex (95% confidence interval (CI) (−2.9; −0.9), *p* < 0.001) and age (95% CI (0.01; 0.11), *p* = 0.013) but not for BMI (95% CI (−0.10; 0.08), *p* = 0.888).

**Figure 2:**
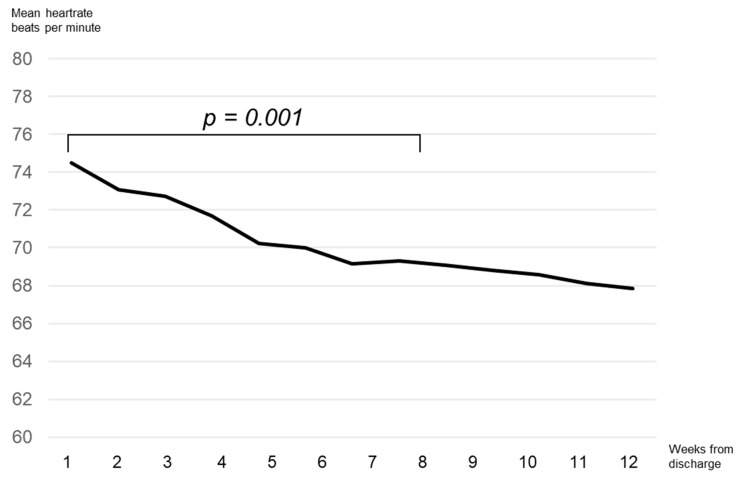
Mean heart rate after discharge.

**Figure 3:**
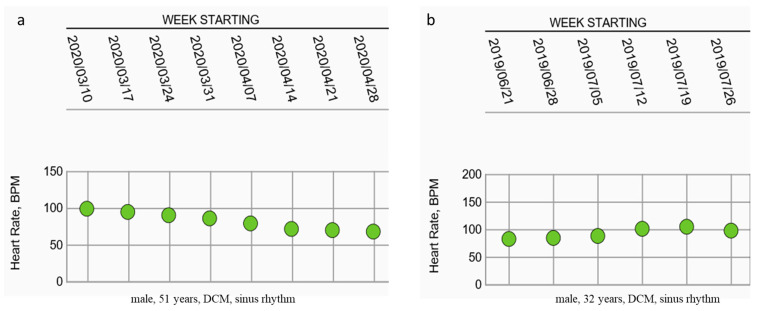
Examples of heart rate control with (**a**) good rate control and (**b**) heart rate overt time significantly above 70 bpm; Abbreviations: DCM, dilated cardiomyopathy; bpm, beats per minute.

Heart rate was documented on the last day of hospital stay and subsequently as a mean value per week. For patients in sinus rhythm with an LVEF of less than 35%, the mean heart rate on the last day of hospital stay was 74.49 ± 13.55 bpm. During the follow-up period, a significant decrease in mean heart rate was observed at 4 (70.24 ± 14.21 bpm, *p* = 0.012), 8 (69.09 ± 10.92 bpm, *p* = 0.001), and 12 (67.83 ± 10.91 bpm, *p* < 0.001) weeks compared with the index value at discharge, as shown in Figure 2. Figure 3 illustrates two examples of two male patients with sinus rhythm with DCM. Figure 3a shows a patient with a good outpatient rate control, whereas Figure 3b shows a patient with an average heart rate of about 100 bpm. When comparing the patients with sinus rhythm after 8 weeks with a heart rate above and under 70 bpm, we found no significances concerning sex, BMI, initial or follow-up LVEF, initial WCD indication, or use of ivabradine at the time of discharge. However, patients with a heart rate of more than 70 bpm were significantly younger (50.40 ± 14.45 vs. 57.07 ± 12.35 years, *p* = 0.04).

## 4. Discussion

Our study shows that the compliance of wearing the prescribed WCD varied by age and sex. Females showed significantly better compliance than men, and patients under the age of 50 showed significantly worse compliance compared with the elderly. Multivariate analysis confirmed these findings. Furthermore, our study illustrates the potential of using online networks showing the daily wearing time and the daily or weekly heart rate of the patient, which allows the treating physician to contact the patient and modify the medication during follow-up. The network offers tailored email notifications in case of a lack of compliance or defined arrhythmic events.

The 2021 published guidelines for diagnosing and treating acute and chronic heart failure recommend WCD with an IIb indication for patients with heart failure and specific risk of SCD [4,22]. These recommendations are mostly based on registry studies and the randomized VEST trial [13,14]. WCD is mainly understood as a bridge to recovery or a bridge to the implantation of a device. In our study, most of the patients did not need an ICD or CRT-D after follow-up, which is congruent with many other studies [23].

Most studies show a daily WCD wearing time of more than 20 h per day, except the VEST trial, where this time was significantly below 20 h per day and where most deaths occurred in patients who did not wear the WCD. Compared with the data of investigators from other WCD-registers (WEARIT-France, median wear time 23.4 h per day [9]; WEARIT-II, median wear time 22.5 h per day [10]; WEARIT-II-EUROPE, mean wear time 20.3 ± 4.6 h per day [11]), this seems to be a standard value for the overall wearing compliance. In their study, Goldenberg et al. found sex differences showing women having a higher burden of atrial and ventricular arrhythmias [24]. They also found better compliance in women than in men, as observed in our study. Garcia et al. reported similar results from the WEARIT-France trial, showing that younger age was the only factor that could be associated with a significant reduction in compliance in a multivariable analysis (odds ratio (OR) 0.97, 95% confidence interval (CI) 0.95–0.99; *p* < 0.01) [9]. Significantly lower wearing compliance in younger patients was also reported by Zylla et al. from their evaluation of 106 WCD prescriptions [25]. In the analysis of Olgin et al., younger age also tended to be associated with lower wearing compliance, though the corresponding uni- and multivariate analysis did not reveal a statistical significance [26]. Reasons for the lower compliance in younger patients are speculative and presumably multifaceted. Factors could include a higher degree of physical activity (that might be impaired by wearing a device) or lower awareness of the overall situation. Hence, in these specific cohorts, the patients have to be educated precisely. Here, the supplier’s online network might be a helpful tool for monitoring the daily wearing time and for contacting the patients in case of noncompliance. Concerning BMI, we found a trend to inferior compliance in obese patients, which could not be found in multivariate analysis. This may be due to the sample size, as in our experience, obese patients tend to show inferior compliance.

In recent years, telemonitoring systems have gained more relevance for the long-term treatment of chronic diseases, such as heart failure [27,28], whereas the significance of benefits, such as an improvement in outcomes or a lower rate of all-cause deaths, is still controversial [29,30,31]. To reduce mortality, current guidelines recommend beta-blockers, ACE-I, AT, AT-neprilysin inhibitors, mineralocorticoid receptor antagonists, and sodium-dependent glucose transporter (SGLT2) inhibitors for all patients with stable, symptomatic heart failure with reduced LVEF [4]. For patients in sinus rhythm with an LVEF < 35% who are still symptomatic despite optimized therapy, the addition of ivabradine is recommended to help them achieve a heart rate lower than 70 bpm [7]. In addition to its primary function as a device to identify and treat life-threatening arrhythmias, a WCD can be used to monitor physiological parameters. For example, Burch et al. found that patients’ physical activity, assessed by step-counts, before the event of ventricular arrhythmias was reduced [32]. In combination with the online platform, the system can be used for telemonitoring purposes. We assessed medical therapy and heart rate at discharge and heart rate course for up to 12 weeks after discharge. Alapati et al. reported discharge heart rate after myocardial infarction and long-term mortality among 6500 patients. They found that a higher discharge heart rate was associated with the three-year mortality and that this risk could be modified by beta-blockers [33]. We observed a significant heart rate reduction during this observational period. Furthermore, we found that patients with a heart rate above 70 bmp at 8 weeks after discharge were significantly younger. This may potentially be explained by inferior compliance. In addition to the use of beta-blockers, the reduction in the mean heart rate may also be due to the effects of an improvement of LVEF under heart failure medication and reduced or restricted activity of the patients wearing the WCD. Due to the retrospective nature of this study and the fact that follow-up consultations within the first 3 months after discharge were mostly carried out by general practitioners or outpatient cardiologists, we have no information about adjustments of the medical therapy (including beta-blockers, ivabradine, or other agents). However, since WCD is mainly prescribed in patients with highly impaired LVEF, we assume that integration of the WCD telemonitoring system in heart failure units and existing follow-up strategies may help increase patient compliance and may give physicians an opportunity to adjust medical therapy. Here, further prospective and randomized studies should be conducted.

Telemonitoring systems not only for WCD but also for other devices (ICD, pacemaker, event recorders, and wearables) are rapidly gaining more importance and attention, generating important and topical data for the treating physicians. The latter are confronted with an excessive mass of data generated every day, which have to be analyzed during daily work. Here, the development of artificial intelligence applications in view of analysis and preselection may play an important role in the near future.

## 5. Limitations

This study has several notable limitations due to its retrospective nature. Furthermore, it was a single-center and non-randomized study. We can only provide retrospective follow-up data of 173 patients presenting to our outpatient clinic. As already mentioned, during the period from discharge to scheduled follow-up, patient care was carried out by outpatient cardiologists and general practitioners who did not have access to the online network. Furthermore, we only received retrospective access to the network. The initial and follow-up LVEF was documented with either MRI or TTE. These LVEF values are not exactly comparable, particularly when a patient did not receive the same method at baseline as at follow-up. As we only included patients between 2016 and 2020, those patients were not treated according to the 2021 heart failure guidelines but were instead treated according to their precursors [7].

## 6. Conclusions

In addition to its function in preventing SCD in a selected cohort, a WCD may be used as a telemonitoring system. Specific patient groups are at risk with regard to compliance and monitoring of heart rate and may benefit from this telemonitoring system, which allows the treating physician to contact the patient, to improve compliance, and to adjust the current medical strategy.

## Figures and Tables

**Table 1:** Baseline characteristics, described by frequencies (*n*, %) or mean and standard deviation (first line) and median and interquartile range (second line).

	Total *n* = 173	Group 1 (*n* = 79; 45.66%)	Group 2 (*n* = 65; 37.57%)	Group 3 (*n* = 29; 16.76%)
Sex (female)	45 (26.01%)	17 (21.52%)	16 (24.62%)	12 (41.38%)
Age (years)	56.64 ± 14.46 58.0 (50.0−67.0)	60.42 ± 11.28 60.0 (53.0−69.0)	54.51 ± 14.17% 57.0 (43.0−66.0)	51.1 ± 19.5 54.0 (38.0−64.0)
Initial LVEF (%)	28.88 ± 10.45 28.0 (22.0−32.0)	29.16 ± 7.8 30.0 (24.0−33.0)	23.57 ± 5.58 23.0 (20.0−29.0)	40.03 ± 15.68 35.0 (28.0−55.0)
BMI	28.57 ± 7.10 27.4 (25.0−31.0)	28.2 ± 5.1 28.0 (31.0−44.0)	30.53 ± 8.9 27.9 (25.0−35.0)	25.27 ± 6 26.0 (24.0−28.0)
NYHA class I NYHA class II NYHA class III NYHA class IV	15 (8.67%) 51 (29.48%) 64 (36.99%) 43 (24.85%)	6 (7.6%) 32 (40.5%) 5 (31.65%) 16 (20.25%)	1(1.54%) 15 (23.97%) 30 (46.15%) 19 (29.23%)	8 (27.59%) 4 (13.79%) 9 (31.04%) 8 (27.58%)
Coronary artery disease	99 (57.23%)	79 (100%)	15 (23.07%)	6 (20.69%)
Arterial hypertension	100 (57.80%)	60 (75.95%)	30 (46.15%)	10 (34.48%)
History of stroke	19 (10.98%)	8 (10.12%)	8 (12.3%)	3 (10.34%)
Diabetes mellitus II	44 (25.43%)	26 (32.91%)	13 (20%)	5 (17.24%)
Sleep apnea	18 (10.40%)	8 (10.13%)	10 (15.38%)	0
COPD	23 (13.29%)	16 (20.25%)	4 (6.15%)	3 (10.34%)
AF	51 (29.48%)	20 (25.32%)	23 (35.38%)	8 (27.59%)
Creatinine at admission (mg/dL)	1.06 ± 0.46 0.95 (0.80−1.16)	1.13 ± 0.59 0.94 (0.81−1.30)	1.04 ± 0.29 1.00 (0.82−1.18)	0.87 ± 0.23 0.09 (0.80−1.00)
Pro-nt-BNP at admission (pg/mL)	4830.39 ± 6657.95 3009 (1470−6198)	5675.43 ± 8994.82 3174 (2046−5899)	4357.09 ± 4102.8 3124 (1170−6274)	3732.74 ± 3856.7 2183 (650−7189)

Abbreviations: LVEF, left ventricular ejection fraction; BMI, body mass index; NYHA, New York Heart association; COPD, chronic obstructive pulmonary disease; AF, atrial fibrillation; BNP, brain natriuretic peptide.

**Table 2:** Medication at discharge.

	*n* = 173	Group 1 (*n* = 79; 45.66%)	Group 2 (*n* = 65; 37.57%)	Group 3 (*n* = 29; 16.76%)
Beta-blocker	168 (97.11%)	79 (100%)	65 (100%)	25 (86.2%)
ACE-I/AT or AT/neprilysin inhibitor	163 (94.22%)	78 (98.73%)	65 (100%)	23 (79.3%)
Aldosteron antagonist	147 (84.97%)	68 (86.07%)	63 (96.92%)	17 (58.62%)
Ivabradin	16 (9.25%)	5 (6.3%)	7 (10.77%)	4 (13.79%)
Digitalis	16 (9.25%)	5 (6.3%)	8 (12.3%)	3 (10.34%)
Anticoagulation	64 (36.99%)	30 (37.97%)	25 (38.46%)	10 (34.48%)

Abbreviations: ACE-I, angiotensin-converting enzyme inhibitor; AT—angiotensin receptor antagonist.

**Table 3:** Follow-up, described by frequencies (*n*, %) or mean and standard deviation (first line) and median and interquartile range (second line).

	*n* = 173	Group 1 (*n* = 79; 45.66%)	Group 2 (*n* = 65; 37.57%)	Group 3 (*n* = 29; 16.76%)
LVEF after follow-up	38.94 ± 10.64 38.0 (30.0−47.0)	37.72 ± 9.68 37.0 (31.0−44.0)	35.81 ± 8.46 36.0 (30.0−43)	49.21 ± 11.59 50.0 (45.0−55.0)
ICD or CRT-D implantation	46 (20.24%)	21 (26.58%)	18 (27.69%)	6 (20.69%)
LVAD or HTX	1 (0.58%)	0	1 (1.54%)	0
VT + shock	1 (0.58%)	1 (1.26%)	0	0
VT ± Response Button, no shock	5 (2.89%)	0	1 (1.54%)	4 (13.79%)
Non sustained VT > 10 s	3 (1.73%)	3 (3.79%)	0	0
Mean daily wearing time (h)	21.19 ± 4.65 23.4 (20.5−23.7)	21.95 ± 3.2 23.4 (21.8−23.8)	20 ± 5.85 23.0 (18.7−23.7)	21.81 ± 4.48 23.4 (22.6−23.7)
Mean WCD wearing time (days)	59.75 ± 35.6 55.0 (34.0−85.0)	54.57 ± 37.19 47.0 (29.0−71.0)	63.08 ± 35.4 64.0 (38.0−89.0)	66.14 ± 30.06 59.0 (48.0−90.0)

Abbreviations: LVEF, left ventricular ejection fraction; ICD, implantable cardioverter defibrillator; CRT-D, cardiac resynchronization therapy defibrillator; LVAD, left ventricular assist device; HTX, heart transplantation; VT, ventricular tachycardia; h, hours; WCD, wearable cardioverter-defibrillator.

## Data Availability

All data are presented in this manuscript.
